# Bone marrow mesenchymal stem cell-derived exosomes alleviate DSS-induced inflammatory bowel disease in mice through inhibiting intestinal epithelial cell pyroptosis via delivery of TSG-6

**DOI:** 10.3389/fimmu.2025.1601591

**Published:** 2025-06-30

**Authors:** Bihua Wu, Shuangyan Su, Xianyu Wang, Yuwei Li, Zhenghao Mei, Fenglin Lou, Le Guo

**Affiliations:** ^1^ Department of Medical Microbiology and Immunology, School of Basic Medical Sciences, Dali University, Dali, Yunnan, China; ^2^ Department of General Surgery, School of Clinical Medicine, Dali University, Dali, Yunnan, China

**Keywords:** inflammatory bowel disease, bone marrow mesenchymal stem cells, exosomes, pyroptosis, intestinal epithelial cells, TSG-6

## Abstract

**Background:**

Inflammatory bowel disease (IBD), characterized by chronic intestinal inflammation and epithelial barrier dysfunction, remains a therapeutic challenge due to the limitations of current treatments, including drug resistance and invasive surgical risks. Emerging evidence implicates intestinal epithelial cells (IECs) pyroptosis as a key contributor to IBD progression. Bone marrow mesenchymal stem cell exosomes (BMSCs-Exo) exhibit anti-inflammatory and tissue-reparative potential, yet the role of tumor necrosis factor-stimulated gene 6 (TSG-6), a critical anti-inflammatory mediator in BMSCs-Exo, in modulating pyroptosis and intestinal barrier integrity remains unexplored. This study investigates the role of TSG-6 contained in mesenchymal stem cell-derived exosomes (MSCs-Exo) in alleviating IBD by modulating the pyroptosis signaling pathway in IECs.

**Results:**

In this study, TSG-6-enriched BMSCs-Exo significantly alleviated intestinal inflammation and pyroptosis in murine IBD models. BMSCs-Exo administration reduced NLRP3 inflammasome activation, suppressed Caspase-1-mediated Gasdermin D (GSDMD) cleavage, and decreased pro-inflammatory cytokine release (IL-1β, IL-18). Notably, TSG-6 knockdown in BMSCs-Exo abolished these protective effects, confirming its essential role in blocking the NLRP3/Caspase-1/GSDMD axis. Furthermore, BMSCs-Exo restored intestinal barrier integrity by upregulating tight junction proteins (e.g., ZO-1, occludin) and reducing epithelial permeability. *In vitro* experiments revealed that BMSCs-Exo directly inhibited pyroptosis in IECs, attenuating cell membrane rupture and inflammatory cascade amplification.

**Conclusion:**

This study identifies TSG-6 as a pivotal mediator in BMSCs-Exo that disrupts pyroptosis-driven IBD pathogenesis by targeting NLRP3 inflammasome activation. The findings highlight BMSCs-Exo as a cell-free therapeutic strategy to mitigate intestinal inflammation and barrier damage, offering advantages over traditional MSCs-based therapies in safety and specificity. By elucidating the TSG-6/NLRP3 regulatory axis, this work provides a novel framework for developing exosome-engineered treatments for IBD and other pyroptosis-related inflammatory disorders.

## 1 Introduction

Inflammatory bowel disease (IBD), a group of chronic relapsing inflammatory disorders primarily comprising ulcerative colitis (UC) and Crohn’s disease (CD), has emerged as a major global public health challenge. Contemporary epidemiological surveillance delineates an accelerating global incidence trajectory, particularly in industrialized nations, while Asian regions including China exhibit an alarming annual growth rate. Notably, Asian regions including China have experienced an annual increase in cases, with projections estimating approximately 1.5 million Chinese IBD patients by 2025 (1, 2). The disease pathogenesis arises from a tripartite nexus of genetic predisposition, environmental perturbations, and immune homeostasis disruption, culminating in persistent mucosal inflammation and epithelial barrier compromise (3). Current therapeutic strategies rely predominantly on pharmacological agents, endoscopic interventions, and surgical procedures. However, these conventional approaches exhibit significant clinical limitations, including suboptimal efficacy, substantial adverse effects, and frequent development of drug resistance. Furthermore, population-based studies demonstrate that only 5.4% of Chinese UC patients ultimately undergo surgical treatment, typically initiated approximately 5.7 years post-diagnosis, reflecting markedly more conservative surgical attitudes compared to Western populations (4). These limitations underscore the urgent need for novel therapeutic strategies. Emerging pathomechanistic insights implicate pyroptosis—a programmed pro-inflammatory cell death mechanism—as a central orchestrator of IBD pathogenesis. This inflammasome-driven process unfolds through sequential NLRP3 activation, caspase-1 proteolysis, and gasdermin D (GSDMD) cleavage, effecting dual pro-inflammatory sequelae: (i) IL-1β/IL-18 maturation/secretion and (ii) cytoplasmic content extrusion via membrane pore formation (5, 6). These events drive IBD progression through progressive expansion and intensification of intestinal inflammation. Clinico-pathological correlations reveal that pyroptotic flux amplifies intestinal inflammation through positive feedback loops, manifesting as progressive mucosal erosion and debilitating symptomatology (hematochezia, tenesmus, malnutrition). Crucially, the intestinal epithelial barrier, composed of tightly connected IECs, plays a critical role in maintaining intestinal homeostasis through its structural and functional integrity. Pyroptosis activation in IECs induces cellular swelling and subsequent rupture, disrupting epithelial continuity. This process is accompanied by altered expression and localization of tight junction proteins (e.g., ZO-1, occludin), ultimately impairing barrier function and causing a marked increase in intestinal permeability (7). These observations posit IECs pyroptosis inhibition as a strategic therapeutic axis for barrier restitution.

Mesenchymal stem cells (MSCs), characterized by robust self-renewal capacity and multilineage differentiation potential, have been identified in multiple human tissues including bone marrow, umbilical cord, placenta, amniotic fluid, and adipose tissue (8). Recent advances highlight the therapeutic potential of MSCs in inflammatory and autoimmune disorders, attributed to their immunomodulatory properties and secretion of bioactive molecules that facilitate tissue repair and mitigate inflammatory response (9–11). Among these secretory components, exosomes—nanoscale extracellular vesicles (30–150 nm) carrying proteins, nucleic acids, and lipids—have garnered significant attention. These vesicles mediate critical intercellular processes including cellular communication, migration, angiogenesis, and tumorigenesis, positioning them as key targets in biomedical research (12–14). Compared to whole-cell therapies, MSCs-Exo exhibit superior translational advantages, featuring reduced immunogenicity, enhanced circulatory stability, and elimination of cell transplantation-associated risks, thereby emerging as safer and more efficient mediators of intercellular communication (15). Accumulating evidence demonstrates that MSCs-Exo exhibit potent immunomodulatory and tissue-reparative capacities. Mechanistically, MSCs-Exo suppress Th1/Th17 lymphocytes differentiation while promoting regulatory T cell (Treg) expansion, thereby rebalancing immune homeostasis and attenuating inflammatory cascades (16). Notably, studies on adipose-derived MSC exosomes (ASC-Exo) have revealed their capacity to accelerate wound closure and enhance tissue regeneration, underscoring translational potential in cutaneous repair (17). Preclinical investigations in IBD models have further established the therapeutic promise of MSCs-Exo, demonstrating dual effects in mitigating intestinal inflammation and stimulating mucosal regeneration (18, 19). Specifically, BMSCs-Exo modulate the intestinal immune microenvironment by inhibiting inflammatory cell infiltration and activation, consequently ameliorating colitis-associated tissue damage (20, 21). *In vivo* studies using murine IBD models have demonstrated that systemic administration of MSCs-Exo attenuates intestinal inflammation, restores mucosal architecture, and alleviates clinical manifestations including diarrhea and hematochezia (22). These collective findings position MSCs-Exo as a novel cell-free therapeutic paradigm for IBD management.

Our investigation focuses on TSG-6, a pluripotent anti-inflammatory protein secreted by MSCs and immune cells under inflammatory stimuli (23). Mechanistically, TSG-6 exerts dual anti-inflammatory actions: (i) direct interaction with extracellular matrix components to modify inflammatory niches, and (ii) immunomodulation through regulation of immune cell effector functions. These properties have been consistently demonstrated across diverse inflammatory pathologies (24, 25). Specifically, TSG-6 secreted by BMSCs crucially alleviates blood-brain barrier disruption following intracerebral hemorrhage through NF-κB pathway inhibition in activated astrocytes, as demonstrated in murine models (26). In silica-induced acute lung injury models, paracrine TSG-6 from BMSCs suppresses NLRP3 inflammasome activation in pulmonary macrophages, effectively mitigating early-stage pulmonary inflammation (27). Notably, intraperitoneal administration of MSCs-Exo in experimental IBD modulates Th2/Th17 lymphocytes responses within mesenteric lymph nodes, significantly improving survival rates and disease severity. Genetic ablation of TSG-6 in MSCs-Exo completely abrogates these therapeutic benefits, as evidenced by recent mechanistic studies (28). Despite these advances, the precise mechanistic basis by which exosomal TSG-6 regulates intestinal mucosal barrier integrity remains elusive.

Given the readily accessible nature of BMSCs and their sustained proliferative capacity under *in vitro* culture conditions, our research has focused on BMSCs-Exo to elucidate their therapeutic potential in mitigating NLRP3/Caspase-1/GSDMD-mediated pyroptosis of IECs and ameliorating IBD. Based on current research evidence, we hypothesize that TSG-6 encapsulated within MSCs-Exo mitigates Caspase-1-mediated GSDMD cleavage and pyroptosis by interfering with NLRP3 inflammasome activation—a critical pathogenic mechanism underlying IBD. Targeting the NLRP3/Caspase-1/GSDMD signaling axis, MSCs-Exo may establish a novel therapeutic paradigm for modulating intestinal inflammation and promoting tissue repair in IBD management.

## 2 Materials and methods

### 2.1 Isolation and culture of BMSCs

BMSCs were isolated from 4-week-old male C57BL/6J mice following an established protocol (29). Briefly, femurs were aseptically excised and transferred to a sterile biosafety cabinet. The bone marrow cavity was repeatedly flushed with pre-chilled phosphate-buffered saline (PBS) using a 25-gauge syringe until the effluent became translucent. The harvested marrow was mechanically dissociated through gentle pipetting and subsequently plated in culture dishes containing MEM-α medium (Gibco, USA) supplemented with 10% fetal bovine serum (FBS; Gibco, USA). Cells were maintained at 37°C in a humidified 5% CO_2_ incubator, with the initial medium replacement conducted at 48 h post-seeding to remove non-adherent hematopoietic cells. Subsequent medium changes were performed every 48 h until cells reached 80-90% confluence, at which point they were subcultured using 0.25% trypsin-EDTA (Gibco, USA). BMSCs between passages 3–5 were utilized for all experiments.

### 2.2 Data download and analysis

The GSE87466 dataset was downloaded from the NCBI Gene Expression Omnibus (GEO) public database (30), which included mucosal biopsy samples from 87 patients with UC and 21 healthy individuals for RNA extraction and microarray analysis. The GSE160804 Series Matrix File data file was downloaded, and the annotation file was licensed according to GPL20115. A gene expression profile dataset including those of 3 healthy individuals and 3 UC patients was incorporated in total. We utilized the Limma software package to identify the differentially expressed genes (DEGs) between the normal group and the disease group. Subsequently, the ggpubr software package was employed to generate box plots for the comparison of the mRNA expressions of NLRP3, Caspase-1, and IL-1β among patients in the normal group and the disease group. Next, the obtained differentially expressed genes (DEGs) were set with the screening criteria of a P-value < 0.05 and |logFc| > 1. The DEGs were analyzed by Gene Ontology (GO) and Kyoto Encyclopedia of Genes and Genomes (KEGG) pathway analysis to obtain the biological functions and signaling pathways involved in the occurrence and development of the disease (31). To explore the impact of DEGs on the pathways in the disease group, single-gene Gene Set Enrichment Analysis (GSEA) was subsequently conducted, with the KEGG signal transduction pathways taken as the predetermined gene sets, and the enrichment of genes therein was detected through the clusterProfiler package (32).

### 2.3 Extraction and identification of BMSCs-Exo

BMSCs exhibiting optimal growth and at approximately 80% confluence at passages P3–P5 were selected. The cells were washed twice with PBS and then cultured in exosome-free serum complete medium. After 48 h, the cell supernatant was collected and sequentially centrifuged at 300 g for 10 min, 2,000 g for 10 min, and 10,000 g for 30 min at 4°C to remove dead cells, organelles, and other impurities. The supernatant was transferred carefully into a 50 mL ultracentrifuge tube and subjected to ultracentrifugation at 100,000 g for 70 min at 4°C. The supernatant was discarded, and the pellet was washed with PBS before being centrifuged again under the same conditions. After discarding the supernatant, the pellet was resuspended in an appropriate volume of PBS. The total protein concentration in the exosomes was determined using a BCA assay and the samples were stored at -80°C (33). The morphology of the exosomes was subsequently examined using transmission electron microscopy. Following appropriate dilution of BMSCs-Exo with PBS and thorough mixing, 10 μl of the exosome solution was loaded onto a copper grid and allowed to stand at room temperature for 2–3 min. Excess liquid was gently removed with clean filter paper, and the exosomes were restained with 3% phosphotungstic acid for 1–2 min. After drying, the grid was examined under a microscope, and appropriate fields of view were selected for imaging and documentation. The exosome surface markers (TSG101, CD81 and CD63) were detected by Western blot.

### 2.4 IECs culture and treatment

The human colon cancer epithelial cell line Caco-2 was purchased from Wuhan Pricella Biotechnology Co., Ltd. (Wuhan, China). The cells were cultured in Dulbecco’s Modified Eagle Medium (DMEM, Gibco) supplemented with 20% fetal bovine serum (FBS, Gibco) and 1% penicillin/streptomycin at 37°C in a humidified atmosphere containing 5% CO_2_. To model inflammation induced by IBD, Caco-2 cells were seeded into 6-well plates at a density of 1 × 10^6^ cells/mL. After cell adhesion, lipopolysaccharide (LPS, 100 μg/mL, Sigma, USA) was added to induce inflammation. For exosome treatment, the adherent cells were further cultured with fresh medium containing different concentrations of exosomes (100 μg/mL, 200 μg/mL, 400 μg/mL) for 48 h, respectively.

### 2.5 Cell viability assay

IECs were seeded into 96-well plates at a density of 5 × 10⁴ cells per well and then placed in a cell incubator for culturing. After cell adhesion, the cells were treated with 100 μg/mL LPS. For exosome treatment, adherent cells were further cultured in fresh medium containing different concentrations of exosomes (100 μg/mL, 200 μg/mL, 400 μg/mL) for 24 h. Following treatment, 10 μL of Cell Counting Kit-8 (CCK-8) reagent (Biosharp) was added to each well. After incubation for 30 min, the absorbance was measured at 450 nm.

### 2.6 Cell transfection

TSG-6 siRNA (siTSG-6), obtained from Universal Biologics, was used to knock down the expression of TSG-6 in BMSCs. After 24 h, siTSG-6 or negative control siRNA (con siRNA) was transfected into BMSCs using Lipofectamine 8000 (Beyotime) according to the manufacturer’s instructions. The efficiency of siRNA transfection was monitored using Western blot analysis. The conditioned medium was collected to isolate exosomes as described earlier. Western blotting was then performed to assess the expression level of TSG-6 protein in the exosomes.

### 2.7 Exosomes labeling and uptaking by IECs

Exosomes were labeled using PKH26 (Umibio) according to the manufacturer’s protocol. Briefly, 10 μL of PKH26 dye working solution was added to 200 μg of exosomes and incubated at room temperature for 10 min. An appropriate amount of PBS was added and mixed, and the labeled exosomes were ultracentrifuged at 100,000 g for 70 min, and resuspended in PBS for use. Then the PKH26-labeled exosomes were incubated with Caco-2 cells at 37°C for 24 h. Cells were washed with PBS, fixed with 4% paraformaldehyde for 30 min, washed with PBS and permeabilized by adding 0.5% Triton X-100 at room temperature for 15 min, and the nuclei of the washed cells were stained with DAPI (Meilunbio), and observed under a fluorescence microscope.

### 2.8 Animal model and treatment

A total of 15 male C57BL/6J mice (aged 8 weeks; weighing 20–22 g) were purchased from SPF (Beijing) Biotechnology Co., Ltd. The mice were housed in individual ventilated cages with the temperature controlled at (23 ± 1°C), humidity controlled at (45-55%) under a 12-hour light/dark cycle, and provided with standard laboratory food and water ad libitum. They were acclimatized for at least one week. The mice were randomly divided into three groups (n = 5 per group): the control group, the dextran sulfate sodium (DSS)-induced mouse colitis group (DSS), and the bone marrow mesenchymal stem cell-derived exosome treatment group (BMSCs-Exo). During the experiment, acute colitis was induced by orally administering double-distilled water containing 2.5% DSS for 7 consecutive days, while the mice in the control group received double-distilled water without DSS. Both the DSS group and the BMSCs-Exo treatment group continuously drank double-distilled water containing 2.5% DSS, and this was replaced with double-distilled water without DSS after 7 days. The BMSCs-Exo treatment group was intraperitoneally injected with BMSCs-Exo with a total protein dose of 1 mg on days 4, 6, and 8 for injury intervention, while the other groups received the same volume of phosphate-buffered saline (PBS).

The body weights of the mice were measured at the same time every morning. Their living conditions, fecal characteristics were observed, and the disease activity index (DAI) was estimated according to the international assessment index (34). On the 10th day, the mice were sacrificed and the colorectal tissues were extracted for further experiments. All animal care and experimental procedures were in line with the guidelines of the Animal Welfare and Ethics Committee of Dali University (Dali, China; approval number: 2024-PZ-153).

### 2.9 Immunohistochemistry

The expression and distribution of proteins related to the classical pyroptosis pathway and tight junction proteins in the colon tissues were analyzed by immunohistochemical staining. Part of the mouse colon tissue was fixed in 4% paraformaldehyde and then embedded in paraffin. After dewaxing, the slides were stained with hematoxylin and eosin (H&E). As described in previous studies, the tissue sections were rehydrated and endogenous peroxidase was quenched with 3% hydrogen peroxide. Subsequently, the slides were incubated overnight at 4°C with anti-NLRP3 (1:200, Servicebio), anti-Caspase-1 (1:750, Servicebio), anti-GSDMD (1:1000, Servicebio), anti-IL-1β (1:800, servicebio), anti-IL-18 (1:500, Servicebio), anti-ZO-1 (1:500, Servicebio), and anti-occludin (1:50, Servicebio) antibodies. Then, they were incubated with horseradish peroxidase (HRP)-conjugated goat anti-rabbit IgG secondary antibody (1:3000) for 1 h at room temperature. The sections were stained with a 3,3’-diaminobenzidine (DAB) kit for 10 min and counterstained with hematoxylin at room temperature for 3 min. Finally, the images were observed and photographed under an optical microscope. The expression of pyroptosis-related proteins and tight junction proteins was analyzed using ImageJ (35, 36).

### 2.10 Hematoxylin-eosin staining of colon

A segment of colon tissue (approximately 4 mm) was excised, fixed, and dehydrated. After embedding in paraffin, tissue sections were cut at 5 μm intervals using a microtome, mounted onto glass slides, dewaxed, and stained with hematoxylin and eosin (H&E). Histopathological analysis of tissue damage was performed according to the Dieleman criteria (37).

### 2.11 Enzyme-linked immunosorbent assay

The cell-free supernatant was collected, and the levels of IL-1β and IL-18 were measured using Human IL-1β ELISA kit (Beijing 4A Biotech, CHE0001), Human IL-18 ELISA kit (Beijing 4A Biotech, CHE0007), Mouse IL-1β ELISA kit (Beijing 4A Biotech) and Mouse IL-18 ELISA kit (Beijing 4A Biotech). All experimental procedures were carried out according to the manufacturer’s instructions. The absorbance of each well was measured at 450 nm using a microplate reader.

### 2.12 Western blot analysis

Proteins were extracted from IECs using RIPA buffer supplemented with protease and phosphatase inhibitors, and protein concentrations were determined using a BCA assay (Thermo Fisher). The protein extracts were separated by sodium dodecyl sulfate polyacrylamide gel electrophoresis (SDS-PAGE) and subsequently transferred to a PVDF membrane (Merck Millipore). The membranes were blocked with 5% skimmed milk for 2 h at room temperature and then incubated overnight at 4°C with primary antibodies: anti-CD81 (1:1000, Abcam), anti-CD63 (1:1000, Abcam), anti-TSG101 (1:1000, Abcam), anti-TSG-6 (1:200, Santa Cruz Biotechnology), according to the manufacturer’s instructions. Additionally, primary antibodies against NLRP3 (1:1000, Cell Signaling Technology), Caspase-1 (1:1000, Cell Signaling Technology), cleaved Caspase-1 (1:1000, Cell Signaling Technology), Gasdermin D (1:1000, Cell Signaling Technology), ZO-1 (1:1000, Cell Signaling Technology), Occludin (1:1000, Cell Signaling Technology), and GAPDH (1:1000, Cell Signaling Technology) were incubated overnight. On the following day, the membranes were incubated with horseradish peroxidase-conjugated secondary antibodies for 1 h at room temperature. The signals were then detected using Super ECL Plus (US EVERBRIGHT) and a chemiluminescent imaging system (Cytiva, ImageQuant LAS 4000 mini). Quantification was performed using ImageJ software.

### 2.13 Statistical analysis

All experiments were performed in triplicate. Data are presented as mean ± SD. Variance analysis was performed using GraphPad Prism software. The T-test was used for comparison between two groups, while one-way analysis of variance (ANOVA) was employed for comparison among multiple groups. A p-value<0.05 was considered a statistically significant.

## 3 Results

### 3.1 Differential expression profiles of NLRP3-mediated classical pyroptosis pathway molecules in IBD patients versus healthy controls

**Figure 1:**
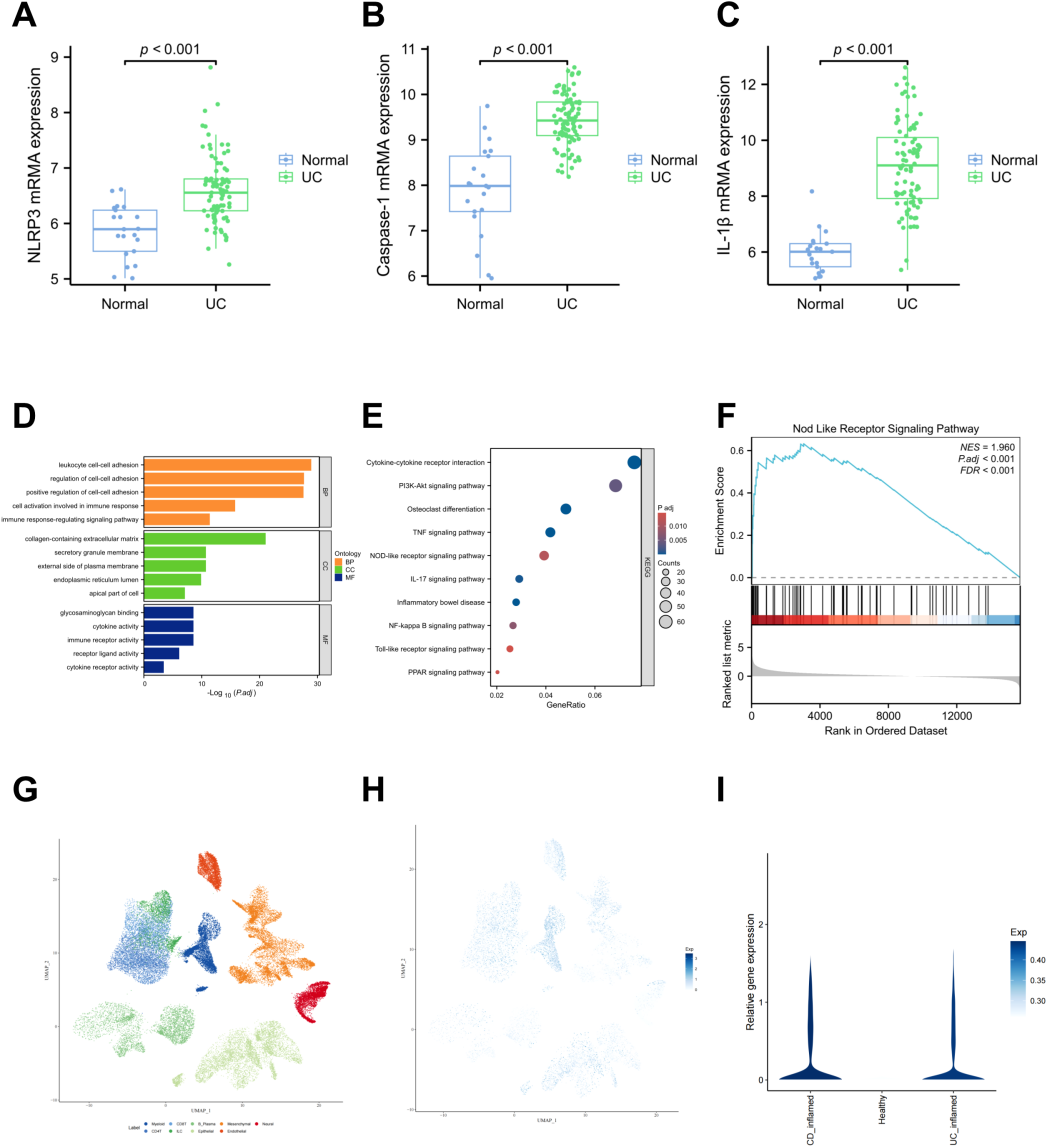
NLRP3、Caspase-1、IL-1β which related to the classical pyroptosis pathway are highly expressed in the colon tissues and IECs of patients IBD and the progression of IBD is associated with the NOD-like receptor signaling pathway. **(A–C)** Comparison of mRNA expression levels of NLRP3, Caspase-1, and IL-1β between UC patients and healthy controls, based on data from the GSE87466 dataset. Perform Gene Ontology (GO) enrichment analysis **(D)**, Kyoto Encyclopedia of Genes and Genomes (KEGG) pathway analysis **(E)** and Gene + Set Enrichment Analysis (GSEA) enrichment analysis **(F)** on the differentially expressed genes from the GSE160804 dataset. **(G)** Cluster the cell populations into nine cell groups. **(H)** The co-expression status of NLRP3, Caspase-1 and GSDMD in the nine cell groups. **(I)** The expressions of NLRP3, Caspase-1 and GSDMD were significantly higher in the UC group and the CD group.

To investigate the differential expression of proteins associated with the classical pyroptosis pathway mediated by the NLRP3 inflammasome between patients with IBD and healthy individuals, we retrieved the GSE87466 dataset from the Gene Expression Omnibus (GEO) database. This dataset includes gene expression profiles from mucosal biopsies of 87 adult patients with moderate to severe active ulcerative colitis and 21 healthy controls. Comparative analysis revealed significantly elevated mRNA levels of NLRP3, Caspase-1, and IL-1β in the colonic mucosal tissues of UC patients compared to healthy controls (**Figures 1A–C**). Subsequently, we obtained the differentially expressed genes between UC patients and healthy individuals from the GSE160804 dataset for GO enrichment analysis and KEGG pathway analysis. The results indicated that the differentially expressed genes were involved in immune cell activation, immune response processes, cytokine activity, and immune receptor activity. Moreover, the KEGG enrichment analysis revealed a significant difference in the NOD-like receptor signaling pathway (**Figures 1D, E**). Therefore, we boldly hypothesized that the NOD-like receptor signaling pathway might be a core pathway influencing IBD. Building on these results, we further performed GSEA. GSEA revealed that the differential gene dataset was enriched at the leading edge of the genes related to the NOD-like receptor signaling pathway, with a relatively high enrichment score and statistical significance (**Figure 1F**). Subsequently, we also utilized the scIBD online single-cell meta-analysis website (38)(http://scibd.cn) to evaluate the expression of proteins related to the classical pyroptosis pathway, specifically NLRP3, Caspase-1, GSDMD, as well as the associations among cells. Firstly, the cell populations were clustered into nine cell groups: Myeloid, CD8^+^ T cells, B/Plasma cells, Mesenchymal, Neural, CD4^+^ T cells, ILCs, Epithelial and Endothelial (**Figure 1G**). Further analysis revealed that NLRP3, Caspase-1, and GSDMD were all expressed in these nine cell groups, among which the expression in the Myeloid and Epithelial groups were relatively more significant (**Figure 1H**). And compared with healthy individuals, UC patients and CD patients exhibit more significant expressions of NLRP3, Caspase-1 and GSDMD (**Figure 1I**). The above results disclose that the NLRP3 inflammasome is significantly activated in the colon tissues and IECs of patients with IBD. This suggests that the classical pyroptosis pathway, mediated by the NLRP3 inflammasome, likely plays a critical regulatory role in the development of IBD.

### 3.2 Isolation and characterization of BMSCs-Exo

**Figure 2:**
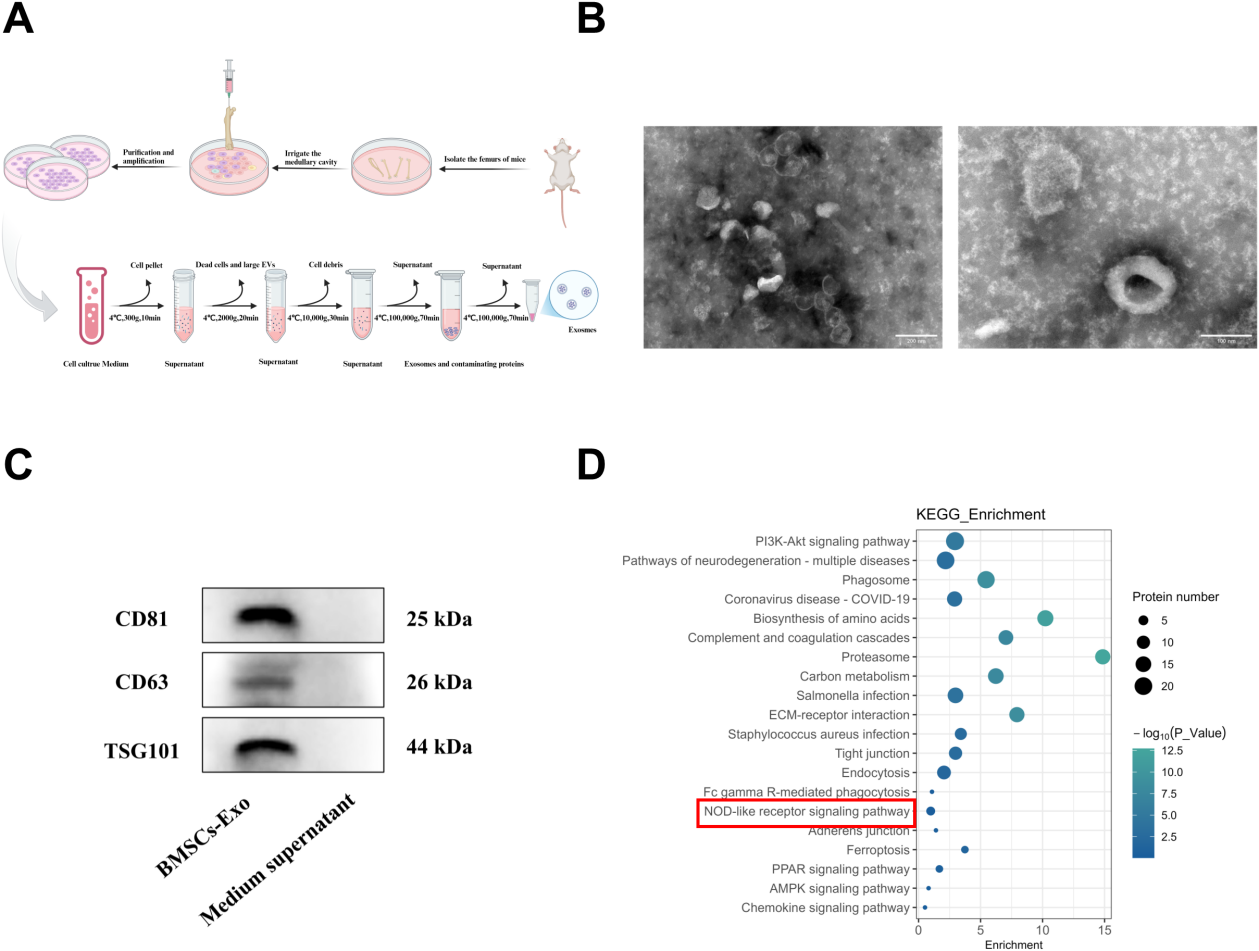
Extraction and characterization of BMSCs-Exo. **(A)** Schematic diagram of exosome isolation via differential ultracentrifugation. **(B)** TEM observation of the BMSCs-Exo morphology (Scale bar = 100 nm and 200 nm). **(C)** Western blot analysis of exosomal markers CD63, CD81, TSG101 in isolated BMSCs-Exo and medium supernatant (centrifugal). **(D)** KEGG pathway enrichment analysis of proteins in BMSCs-Exo. Created in BioRender.

BMSCs were successfully isolated from bone marrow and cultured. Exosomes were subsequently extracted from the culture supernatant by ultracentrifugation (**Figure 2A**), and characterized using transmission electron microscopy (TEM) and Western blot analysis. TEM imaging revealed that the BMSCs-Exo maintained intact vesicular structures, exhibiting characteristic cup-shaped morphology with an average diameter of approximately 100 nm (**Figure 2B**). Western blot analysis further confirmed the exosomal nature of these particles by showing positive expression of conserved exosomal surface markers, including CD81, CD63, and TSG101 (**Figure 2C**). Collectively, these morphological and biochemical characterizations confirmed the successful isolation of exosomes from BMSCs cultures. Substantial evidence has established that exosomes regulate physiological processes in recipient cells by transporting bioactive molecules, including functional proteins and miRNAs, playing a critical regulatory role in tissue regeneration (12, 39, 40). Building upon these findings, we systematically analyzed the protein composition of BMSCs-Exo using mass spectrometry. KEGG pathway enrichment analysis further revealed their enrichment in the NOD-like receptor signaling pathway (**Figure 2D**). These results suggest that BMSCs-Exo may participate in the pathological progression of IBD through modulation of the NOD-like receptor signaling pathway.

### 3.3 BMSCs-Exo protects against acute DSS-induced colitis in mice

**Figure 3:**
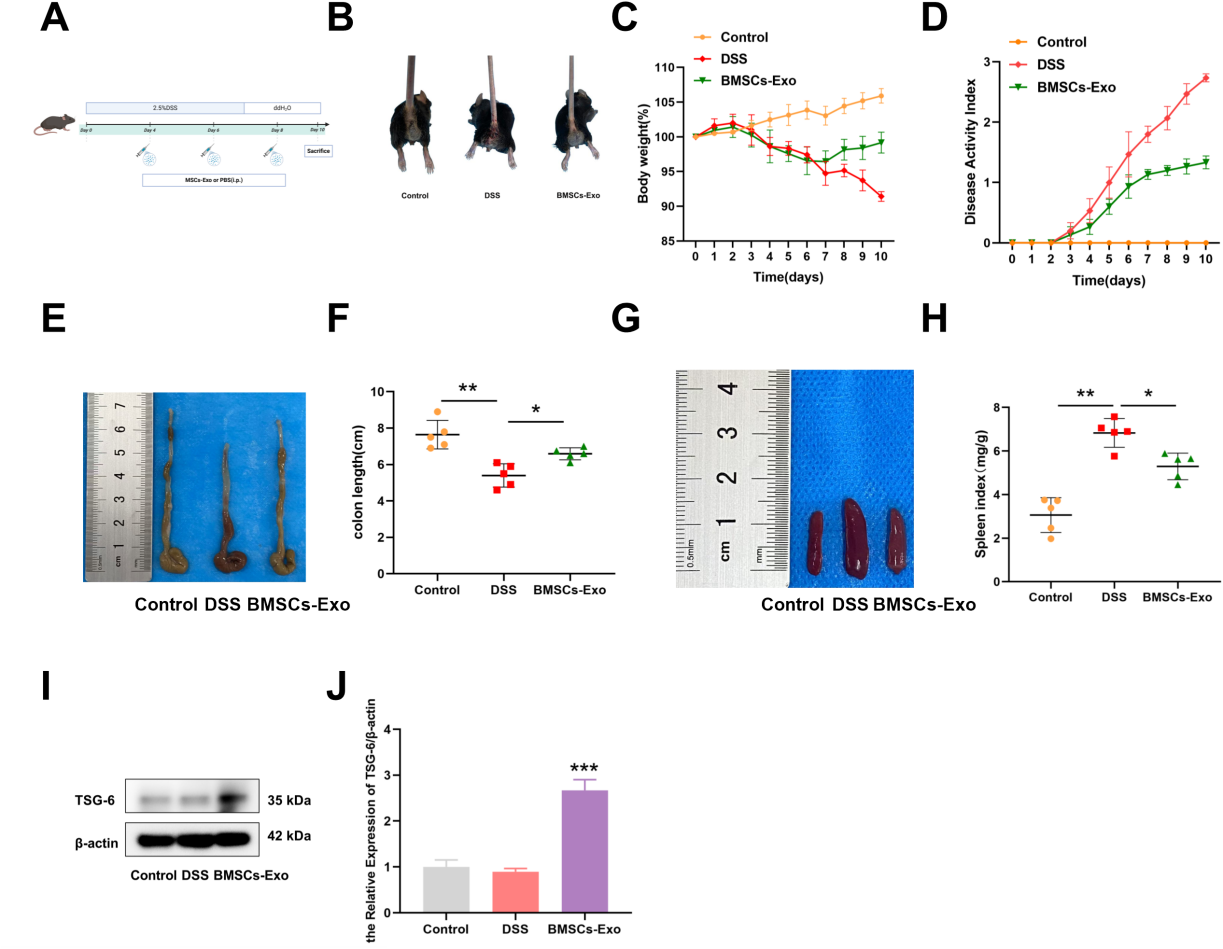
BMSCs-Exo ameliorate DSS-induced acute colitis in mice. **(A)** Schematic diagram of DSS-induced acute colitis and BMSCs-Exo administration protocol. **(B)** Hematochezia. **(C)** Body weight changes. **(D)** Disease activity index combining weight loss, stool consistency, and bleeding scores. **(E)** Macroscopic images of colons and **(F)** quantification of colon length. **(G)** Macroscopic spleen images and **(H)** spleen index. **(I)** Western blot analysis of the protein expression level of TSG-6. **(J)** Quantification of protein levels of TSG-6. *P<0.05, **P<0.01, ***P<0.001. Created in BioRender.

To investigate the protective effects of BMSCs-Exo on inflammatory bowel disease, a murine colitis model was established by oral administration of 2.5% DSS followed by therapeutic intervention with BMSCs-Exo via intraperitoneal injection (**Figure 3A**). Daily monitoring was performed throughout the experimental period to assess body weight variations, diarrhea severity, and fecal occult blood presence. As shown in **Figure 3C**, control group mice exhibited progressive weight gain, whereas DSS-treated mice demonstrated significant weight loss commencing from day 2 post-DSS administration. From day 4 onward, DSS-challenged mice developed characteristic colitis symptoms including persistent diarrhea, hematochezia, reduced food intake, and diminished responsiveness to external stimuli. Concurrently, the disease activity index (DAI) scores in the DSS group showed marked elevation compared to control animals. BMSCs-Exo administration significantly ameliorated diarrheal symptoms and hematochezia, accompanied by a substantial reduction in DAI scores (**Figure 3D**). On day 10, all mice were euthanized for macroscopic evaluation of colonic and splenic morphology. Postmortem analysis revealed significant colonic shortening and splenomegaly in DSS-treated mice, pathological manifestations likely attributable to an overactive immune response. Notably, BMSCs-Exo treatment effectively attenuated these DSS-induced pathological alterations in both colon length and spleen size, demonstrating its therapeutic potential in ameliorating experimental colitis manifestations (**Figures 3E–H**). Interestingly, elevated levels of TSG-6 protein were detected in colonic tissues of mice treated with BMSCs-Exo, suggesting that the therapeutic effects of BMSCs-Exo on inflammatory bowel disease may be mediated through the delivery of TSG-6 protein (**Figures 3I, J**).

### 3.4 BMSCs-Exo alleviates acute colitis in mice via suppression of NLRP3-mediated pyroptosis

**Figure 4:**
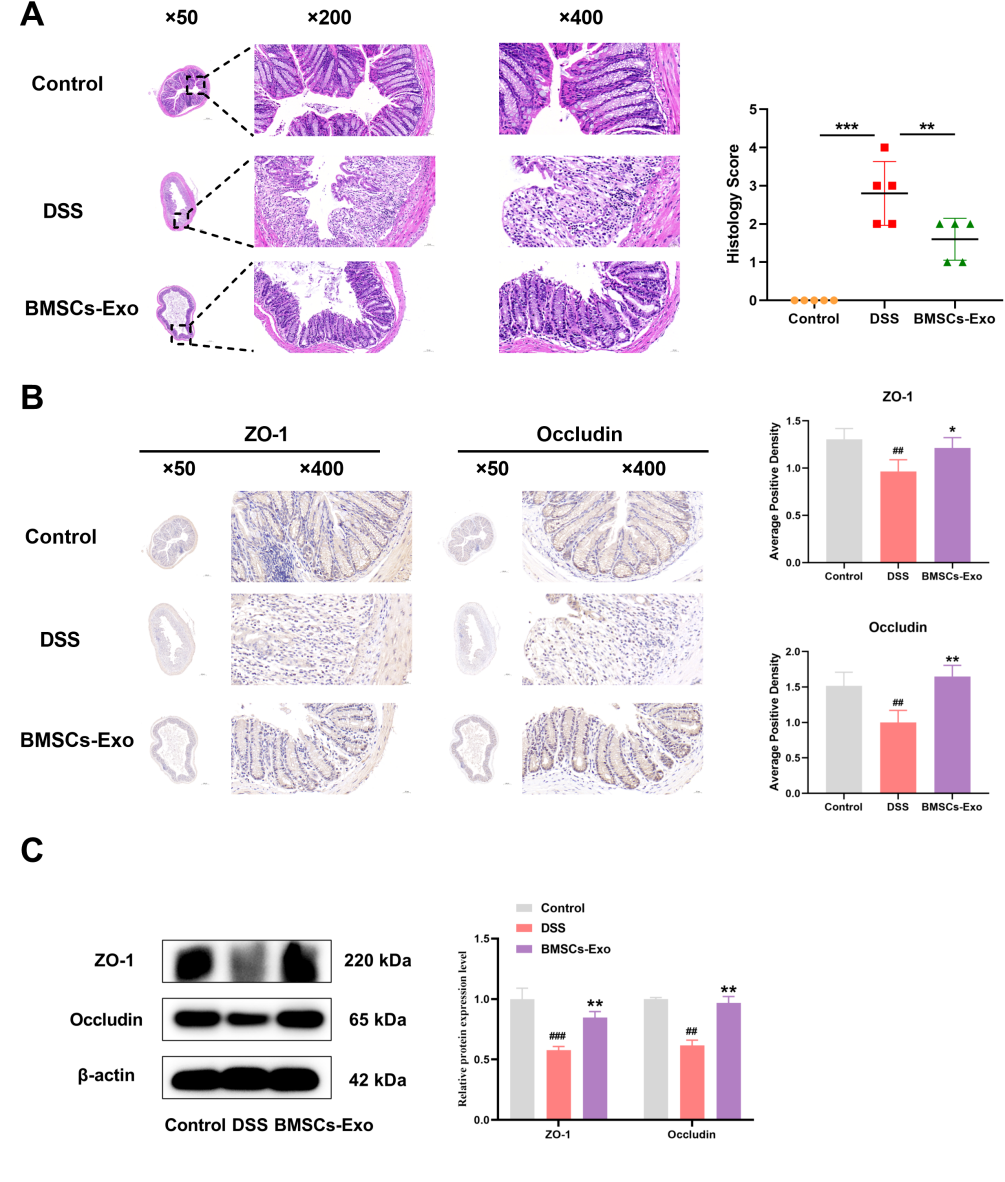
BMSCs-Exo enhance the expression of tight junction proteins, thereby protecting the intestinal mucosal barrier. **(A)** H&E staining of colonic tissues (magnification: ×50, ×200, and ×400) and histological scoring. **(B)** Immunohistochemical staining analysis of ZO-1 and Occludin protein expression in mouse colonic tissues (magnification: ×50 and ×400). **(C)** Western blot analysis of ZO-1 and Occludin protein expression in mouse colonic tissues. ^##^P < 0.01, ^###^P < 0.001 vs Control by ANOVA; *P < 0.05, **P < 0.01, ***P<0.001 vs DSS by ANOVA.

Histopathological analysis was employed to evaluate DSS-induced colonic injury in mice. Compared with the Control group, the DSS group exhibited extensive mucosal erosion, crypt depletion, disruption of mucosal epithelial and glandular structures, and significant infiltration of granulocytes and lymphocytes, as observed through HE staining (**Figure 4A**). In contrast, the BMSCs-Exo treatment group showed reduced colonic damage and intestinal inflammation. Consistent with these findings, histological scoring indicated that pathological changes in the colonic mucosa were significantly alleviated in the BMSCs-Exo-treated group (**Figure 4A**). To assess the impact on intestinal barrier integrity, the expression of tight junction proteins Occludin and ZO-1 in colonic tissues was examined using immunohistochemical staining and Western blot analysis. The results revealed that Occludin and ZO-1 expression levels were markedly decreased in the DSS group compared to the Control group, whereas their expression was substantially restored in the BMSCs-Exo-treated group (**Figures 4B, C**). These observations suggest that BMSCs-Exo enhances the expression of tight junction proteins, thereby protecting the intestinal mucosal barrier.

**Figure 5:**
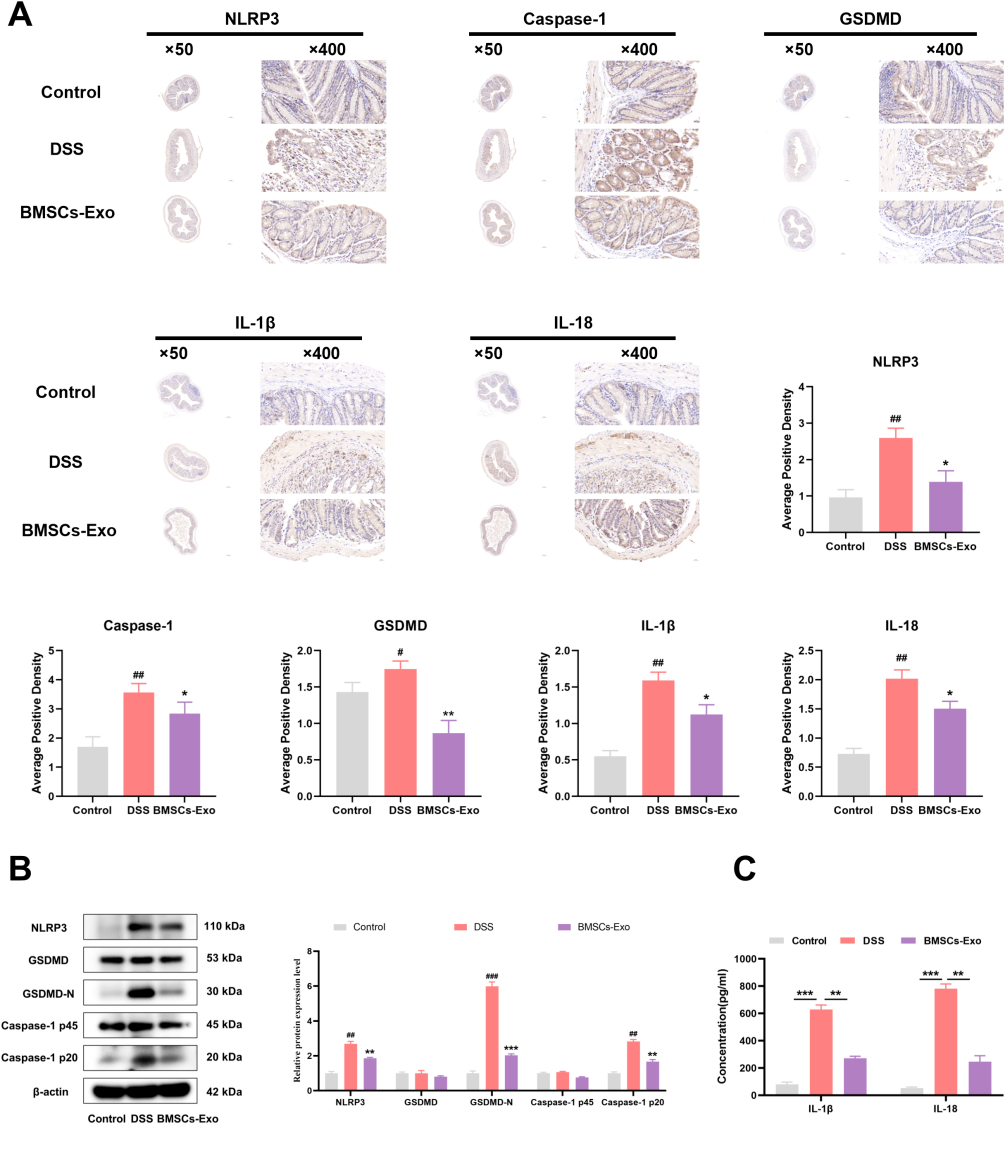
BMSCs-Exo protects against DSS-induced colitis by inhibiting NLRP3-mediated pyroptosis. **(A)** Expression levels of NLRP3, Caspase-1, GSDMD, IL-1β and IL-18 proteins in mouse colon tissues were detected by immunohistochemistry (magnification, ×50 and ×400), and the average positive area was calculated. **(B)** Expression levels of NLRP3, GSDMD, GSDMD-N, Caspase-1 p45, and Caspase-1 p20 in mouse colon tissues were assessed by Western blot. **(C)** Levels of IL-1β and IL-18 in mouse serum were measured by ELISA. ^#^P < 0.05, ^##^P < 0.01, ^###^P < 0.001 vs Control by ANOVA; *P < 0.05, **P < 0.01, ***P<0.001 vs DSS by ANOVA.

To further elucidate the protective mechanisms of BMSCs-Exo, the expression of pyroptosis-related proteins NLRP3, Caspase-1, and GSDMD in colonic tissues was evaluated via immunohistochemical staining. Compared with the expression observed in the IBD group, the BMSCs-Exo-treated group exhibited significantly reduced levels of NLRP3, Caspase-1, GSDMD, IL-1β and IL-18 (**Figure 5A**). Supporting these results, western blot analysis further confirmed that BMSCs-Exo mitigated the DSS-induced upregulation of NLRP3 and the enhanced cleavage of Caspase-1 and GSDMD, effectively normalizing their expression levels (**Figure 5B**). Additionally, serum levels of the pro-inflammatory cytokines IL-1β and IL-18 were significantly lower in the BMSCs-Exo-treated group compared to the DSS group (**Figure 5C**). Collectively, these findings demonstrate that BMSCs-Exo alleviates DSS-induced colitis in mice by suppressing NLRP3-mediated pyroptosis, offering a mechanistic basis for its protective role against intestinal inflammation and injury.

### 3.5 BMSCs-Exo mitigates pyroptosis in IECs through NLRP3 inflammasome suppression *in vitro*

**Figure 6:**
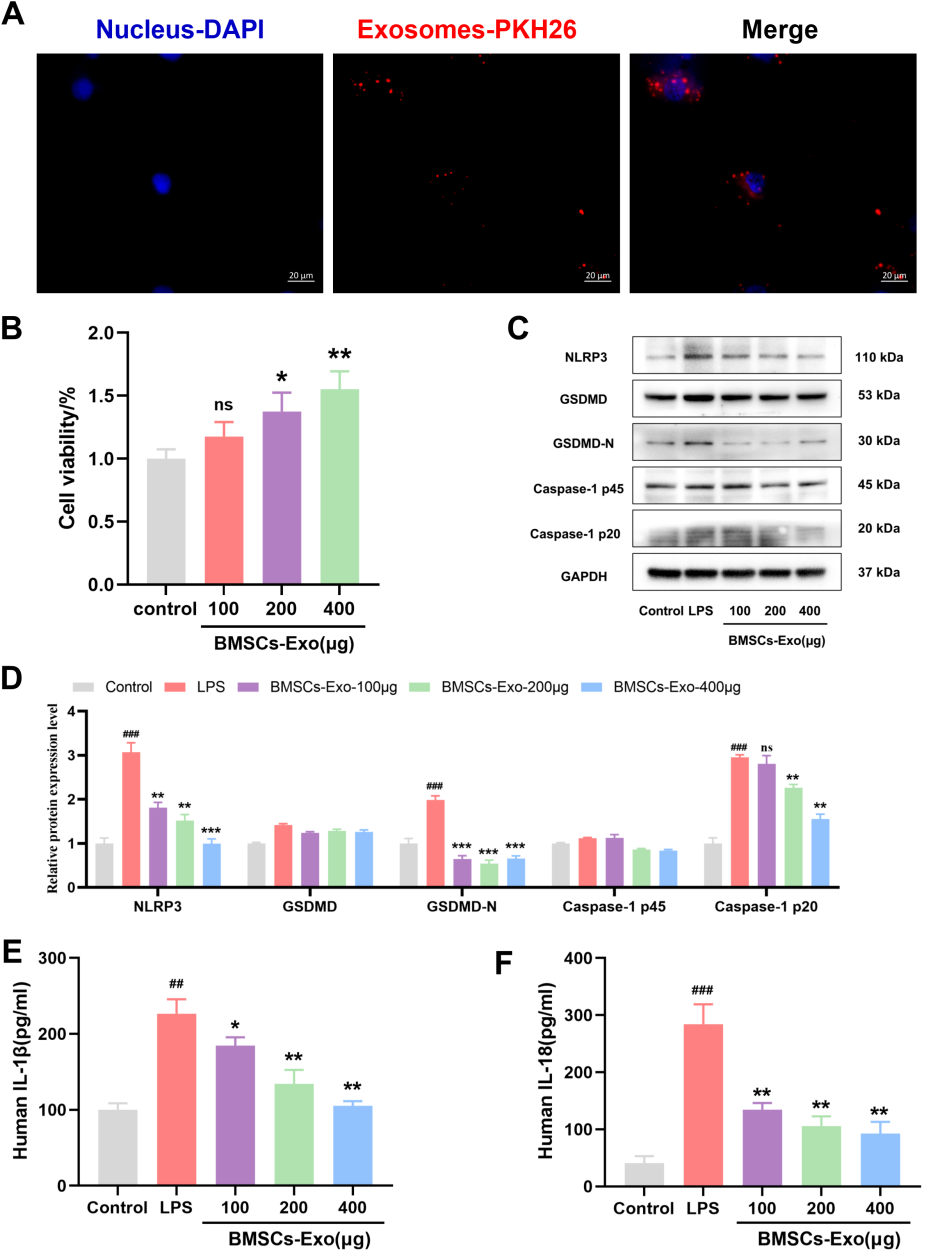
BMSCs-Exo alleviate IBD by inhibit LPS-induced pyroptosis in IECs. **(A)** Fluorescence microscopy analysis of PKH26-labeled BMSCs-Exo internalization by IECs. Scale bars are 20 μm. **(B)** Cell viability was assessed by CCK8 assay. **(C)** Western blot analysis of the protein expression level of NLRP3, GSDMD, GSDMD-N, Caspase-1 and Cleave-Caspase-1 in IECs. **(D)** Quantification of protein levels of NLRP3, GSDMD, GSDMD-N, Caspase-1 and cleave-Caspase-1. **(E)** Analysis the IL-1β and IL-18 expression levels in supernatant of IECs by ELISA. ^##^P<0.01, ^###^P<0.001 vs Control by ANOVA; *P<0.05, **P<0.01, ***P<0.001 vs LPS by ANOVA. ns indicates no statistical significance.

The aforementioned experimental results demonstrate that BMSCs-Exo can alleviate DSS-induced IBD-related symptoms in mice by modulating NLRP3 inflammasome-mediated pyroptosis. Previous studies have shown that enterocyte pyroptosis plays a critical role in the onset and progression of IBD. To further investigate the *in vitro* reparative effect of BMSCs-Exo on IBD, the human colon epithelial cell line Caco-2 was selected. Initially, BMSCs-Exo were labeled with the red fluorescent dye PKH26, followed by co-incubation with IECs. Fluorescence microscope analysis revealed that PKH26-labeled BMSCs-Exo could be well internalized by IECs (**Figure 6A**). Furthermore, IECs were treated with different concentrations of BMSCs-Exo (100 μg, 200 μg, 400 μg) respectively to evaluate its effect on the proliferation ability of intestinal epithelial cells. The CCK-8 assay revealed that BMSCs-Exo promoted IECs proliferation in a concentration-dependent manner (**Figure 6B**). An *in vitro* inflammation model was then established through LPS induction, followed by treatment with BMSCs-Exo. Consistent with the findings from the animal model, Western blot analysis showed that treatment with different concentrations of BMSCs-Exo significantly reduced the expression of NLRP3, GSDMD-N, and cleaved Caspase-1, which were upregulated by LPS induction in IECs (**Figures 6C, D**). Meanwhile, our research results showed that LPS upregulated the expression of IL-1β and IL-18 in IECs. However, compared with the LPS group, BMSCs-Exo counteracted these effects in a dose-dependent manner (**Figures 6E, F**). Overall, these results demonstrate that in the *in vitro* inflammation model, treatment with various concentrations of BMSCs-Exo effectively suppressed NLRP3 inflammasome activation, thereby alleviating pyroptosis in IECs.

### 3.6 BMSCs-Exo containing TSG-6 inhibits NLRP3 mediated pyroptosis in IECs

**Figure 7:**
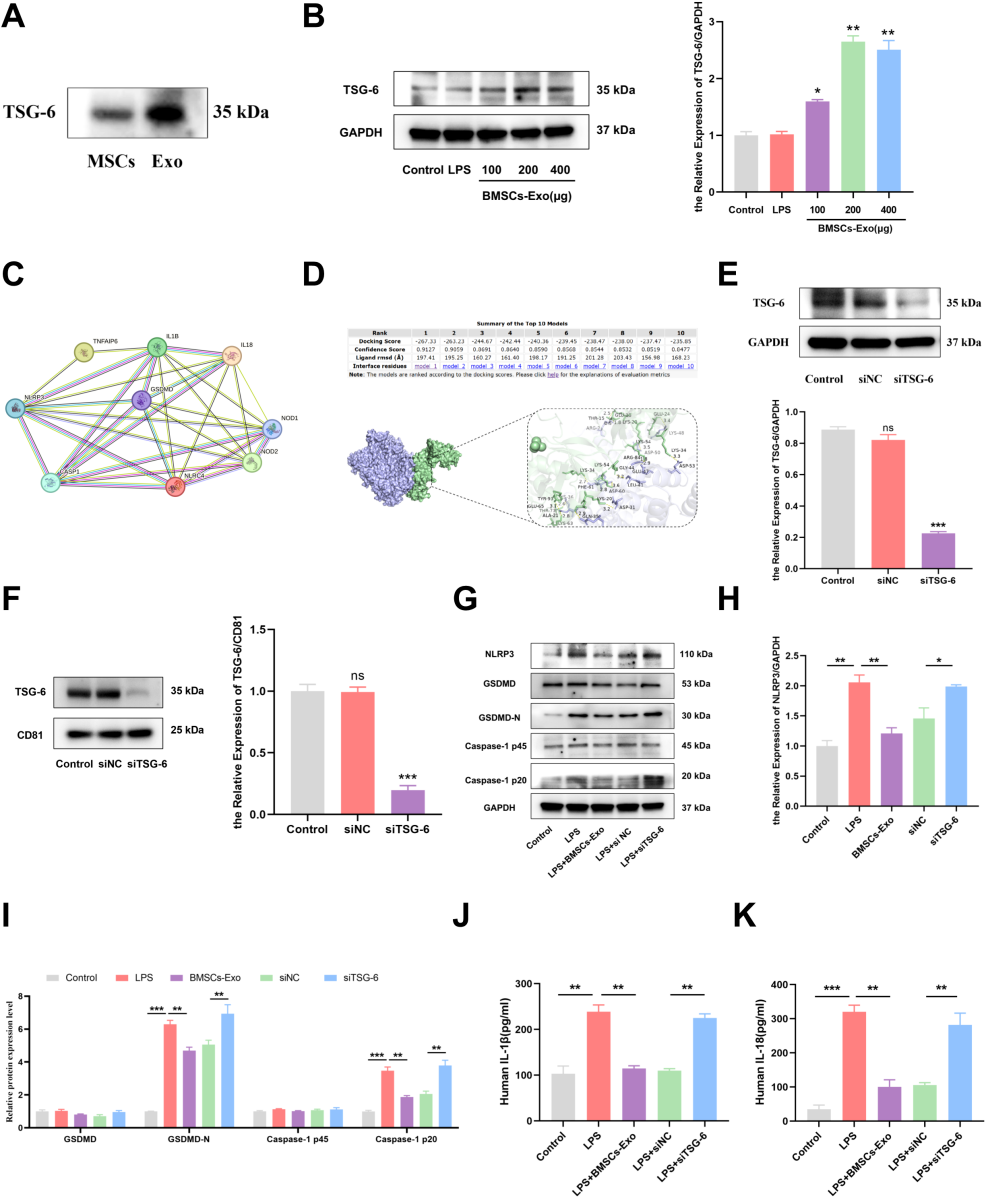
TSG-6 in BMSCs-Exo inhibited LPS-induced pyroptosis of IECs and thus alleviated IBD. **(A)** Western blot analysis of the protein expression level of TSG-6. **(B)** Expression of TSG-6 in IECs was detected by Western blot. **(C)** The protein-protein interaction network between TSG-6 (TNFAIP6) and NOD-like receptor signaling pathway components was constructed using the STRING database. **(D)** The interactivity of TSG-6 and NLRP3 predicated by HDOCK server. **(E)** The expression of TSG-6 in siRNA-transfected BMSCs was detected by Western blot. **(F)** Western blot analysis of TSG-6 expression in exosomes derived from bone marrow mesenchymal stem cells transfected with siRNA. **(G)** Western blot analysis of the protein expression level of NLRP3, GSDMD, GSDMD-N, Caspase-1 and cleave-Caspase-1 in IECs. **(H, I)** Quantification of protein levels of NLRP3, GSDMD, GSDMD-N, Caspase-1 and Cleave-Caspase-1. **(J, K)** Analysis the IL-1β and IL-18 expression levels in supernatant of IECs by ELISA. *P<0.05, **P<0.01, ***P<0.001 by ANOVA.

Previous studies have demonstrated that proteins, nucleic acids, microRNAs, and long non-coding RNAs (lncRNAs) carried by BMSCs-Exo are key mediators of its immunomodulatory effects (41–43). Notably, TSG-6 has been shown to play a crucial role in various inflammatory diseases (44, 45). To investigate whether TSG-6 mediates the inhibition of pyroptosis in IECs by BMSCs-Exo, we first assessed the expression of TSG-6 using western blot analysis. The results revealed that TSG-6 was expressed in both BMSCs and BMSCs-Exo, with a higher enrichment of TSG-6 in BMSCs-Exo when the same amount of protein was loaded for both samples (**Figure 7A**). Subsequently, in an inflammation model, IECs were treated with BMSCs-Exo at concentrations of 100 μg, 200 μg, and 400 μg. Western blot analysis revealed that all tested concentrations of BMSCs-Exo increased TSG-6 protein expression in IECs (**Figure 7B**). A protein-protein interaction network between TSG-6 and NOD-like receptor signaling pathway components was constructed using the STRING database (https://cn.string-db.org) with a minimum interaction confidence score of 0.400. Visualization revealed that TSG-6 exhibited connectivity with key nodes of the NLRP3-mediated pyroptosis pathway. This suggests that TSG-6 may play a critical role in preserving intestinal barrier integrity and attenuating IBD progression through pathway modulation (**Figure 7C**). Molecular docking simulations via the HDOCK server (http://hdock.phys.hust.edu.cn) further predicted specific binding sites between TSG-6 and NLRP3 protein, indicating a potential direct interaction between these molecules (**Figure 7D**). Next, we used small interfering RNA (siRNA) to silence the expression of TSG-6 in BMSCs-Exo. Exosomes derived from BMSCs with TSG-6 knocked down (siTSG-6-Exo) were then obtained from the conditioned medium, and the knockdown efficiency of TSG-6 in BMSCs and BMSCs-Exo was confirmed by western blot analysis (**Figures 7E, F**). Subsequently, we employed western blot to identify whether the inhibition of TSG-6 in BMSCs-Exo affected the pyroptosis of IECs. The results demonstrated that after treatment with BMSCs-Exo, the increased protein levels of NLRP3, Caspase-1 p20 and Gasdermin D-N (GSDMD-N) in lipopolysaccharide (LPS)-induced IECs could be decreased. However, when the expression of TSG-6 in exosomes was knocked down using siRNA, the regulatory function of BMSCs-Exo was reversed (**Figures 7G–I**). The results of ELISA also indicated that the expression levels of IL-1β and IL-18 in the siTSG-6-Exo group were significantly higher than those in the BMSCs-Exo group (**Figures 7J, K**). In conclusion, the above results demonstrate that TSG-6 in BMSCs-Exo can inhibit the pyroptosis of IECs by regulating the NLRP3/Caspase-1 signaling axis, thus clarifying a new mechanism for BMSCs-Exo in the treatment of IBD.

## 4 Discussion

**Figure 8:**
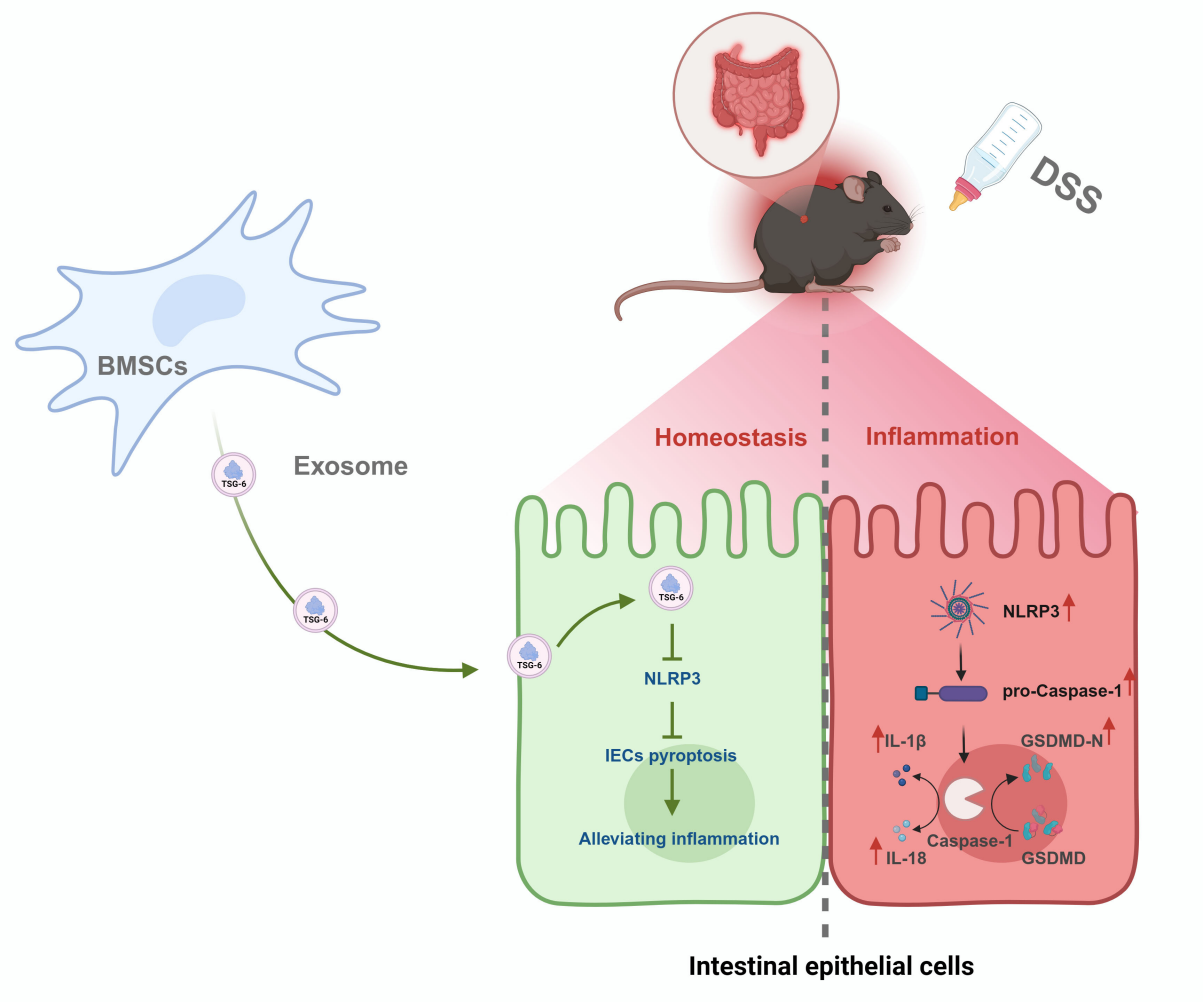
Mechanism of BMSCs-Exo in alleviating DSS-induced IBD via TSG-6-mediated suppression of NLRP3 inflammasome-dependent pyroptosis in IECs.

The current study elucidates a previously unrecognized mechanism through which TSG-6-enriched BMSCs-Exo mitigates pyroptosis-driven intestinal inflammation and epithelial barrier dysfunction in IBD. Our findings establish TSG-6 as a critical mediator in BMSCs-Exo that disrupts the NLRP3/Caspase-1/GSDMD axis, thereby attenuating IECs pyroptosis and restoring mucosal integrity (**Figure 8**). These results advance our understanding of exosome-mediated therapeutic strategies for IBD and position TSG-6 as a pivotal molecular target for modulating pyroptosis in inflammatory pathologies.

The integrity of the intestinal barrier plays a pivotal role in maintaining gut homeostasis. Emerging evidence suggests that its dysfunction has been increasingly recognized as a central component in the pathogenesis of IBD, potentially driving sustained immune activation and inflammatory cascades through compromised mucosal barrier function (46). IECs as the core structural and functional units of the intestinal barrier, play pivotal roles in host defense through their specialized spatial organization and physiological properties. These polarized cells form a tightly regulated monolayer, establishing a dynamic interface that physically segregates the gut microbiota and luminal antigens from the underlying immune compartments. This strategic separation is critical for maintaining intestinal immune homeostasis while coordinating essential defensive responses against luminal threats (47). During pyroptosis, GSDMD protein oligomerizes to form pores in the plasma membrane, resulting in osmotic imbalance, cellular swelling, and eventual lytic cell death (6, 48). This process facilitates the release of pro-inflammatory cytokines (e.g., IL-1β and IL-18) and simultaneously compromises the integrity of the intestinal barrier. The dual effects of pyroptosis play a central role in the pathogenesis of IBD by amplifying immune dysregulation and epithelial damage. Activation of the NLRP3 inflammasome and subsequent Caspase-1-dependent pyroptosis constitute a hallmark of IBD pathogenesis (49, 50). Our data demonstrate that BMSCs-Exo robustly inhibit NLRP3 inflammasome assembly and attenuate Caspase-1-mediated GSDMD activation in IECs. This suppression aligns with previous reports that MSC-derived exosomes modulate inflammasome activity in macrophages and epithelial cells (19, 51). Notably, our study uniquely identifies TSG-6 as the principal effector within BMSCs-Exo responsible for their anti-pyroptotic activity. Genetic ablation of TSG-6 in BMSCs-Exo abolished these therapeutic benefits, underscoring its non-redundant role in targeting the NLRP3/Caspase-1 axis—a distinction from other exosomal components. While previous studies have demonstrated that TSG-6 alleviates DSS-induced colitis symptoms in murine models, the precise molecular mechanisms underlying this protection remained elusive (52). Encouragingly, our findings establish that TSG-6 is selectively packaged into BMSCs-Exo and directly suppresses pyroptosis in IECs. This mechanistic insight positions TSG-6-enriched exosomes as a novel therapeutic modality for IBD, offering enhanced safety and targeted efficacy. Beyond modulating pyroptosis, our study demonstrates that BMSCs-Exo administration restored intestinal barrier integrity through dual mechanisms mediated by upregulating tight junction proteins and reducing epithelial permeability. By suppressing pyroptosis in IECs, BMSCs-Exo maintained epithelial continuity and diminished release of IL-1β and IL-18 from pyroptotic IECs, thereby disrupting the positive feedback loop of inflammation-driven barrier dysfunction. Current therapeutic approaches for IBD, including biologics (e.g., anti-TNFα antibodies such as infliximab and adalimumab) and small-molecule inhibitors (e.g., JAK/STAT pathway blockers like tofacitinib), have significantly improved disease management. However, these treatments are often associated with notable drawbacks, including systemic immunosuppression, increased susceptibility to infections, high treatment costs, and substantial relapse rates following drug withdrawal (53). In contrast, our findings demonstrate that BMSCs-Exo offer a promising alternative with several potential advantages. As cell-free therapeutic agents, BMSCs-Exo retain many beneficial properties of MSCs without the risks associated with live cell transplantation, such as unwanted engraftment, tumorigenicity, or induction of allogeneic immune responses. The absence of MHC molecules and replicative ability in BMSCs-Exo contributes to their favorable safety profile and reduces the likelihood of immune rejection or off-target effects (54). Moreover, unlike conventional therapies that require systemic administration and often result in widespread immunomodulation, BMSCs-Exo can be delivered through non-invasive routes such as oral or rectal administration. Their nanoscale size and lipid bilayer structure facilitate efficient uptake and targeted accumulation in inflamed colonic tissues, enhancing therapeutic precision while minimizing systemic exposure. Compared with biologics and small-molecule drugs, which often require continuous administration and monitoring due to variable patient responses and potential adverse events, BMSCs-Exo represent a potentially safer, more targeted, and cost-effective therapeutic modality. These advantages underscore the translational potential of BMSCs-Exo as a novel therapeutic strategy for IBD, warranting further investigation in clinical settings.

Recent advances in exosome research have highlighted their therapeutic potential in IBD, leveraging their ability to target inflamed tissues and modulate immune responses. However, natural exosomes face significant hurdles in clinical translation, including limited targeting specificity, insufficient payload capacity, and challenges in maintaining stability during storage. To overcome these limitations, innovative bioengineering strategies have emerged as promising solutions. Notably, genetic engineering approaches demonstrate significant potential: co-transfection of human placental mesenchymal stem cells (PMSCs) with silk fibroin-binding peptide (SFBP)-GlucMS2 (SGM) complexes and pac-miR-146a-pac fusion proteins enables precise integration of SGM proteins into the plasma membrane. During exosome biogenesis, miR146a achieves efficient encapsulation via molecular interactions between bacteriophage MS2 capsid proteins and pac sites, with experimental data revealing a 10-fold enhancement in loading efficiency (55). Furthermore, microfluidic delivery systems have been shown to optimize exosome delivery. Electrospray microfluidic technology facilitates the encapsulation of MSCs-Exo within sodium alginate (SA) hydrogel microspheres modified with gelatin interlayers (56). This system not only preserves exosome bioactivity during storage but also protects against harsh gastrointestinal conditions while enabling site-specific controlled release. Importantly, to overcome 5-fluorouracil (5-FU) resistance in colorectal cancer (CRC) treatment, recent studies demonstrate that electroporation-mediated loading of 5-FU into engineered exosomes significantly reverses chemoresistance and enhances therapeutic efficacy, offering a promising strategy for clinical translation (57).

Our findings reveal that TSG-6 within BMSCs-Exo plays a pivotal role in alleviating DSS-induced IBD by inhibiting pyroptosis and upregulating tight junction proteins, thus providing dual protective effects on the intestinal mucosa. These insights not only underscore the significance of TSG-6 as a therapeutic mediator but also highlight the need for future research to refine exosome modifications. Despite these promising developments, the full therapeutic potential of exosomes in IBD remains constrained by unresolved challenges, necessitating further exploration of engineering strategies to enhance their clinical applicability. Specifically, efforts should focus on improving targeting specificity to IECs, increasing TSG-6 payload efficiency, and developing robust storage protocols to ensure long-term stability. Such advancements could bridge the gap between preclinical success and clinical translation, positioning engineered BMSCs-Exo as a safe, effective, and targeted nanotherapeutic option for IBD and other pyroptosis-driven disorders. This study establishes a foundation for these future directions, offering a framework to harness exosome-mediated intercellular communication for precision medicine.

## Data Availability

The datasets generated for this study are available in an online repository. The name of the repository and the accession number are provided in the article. The data are also available from the corresponding author upon reasonable request.
